# Timing Markers of Interaction Quality During Semi-Hocket Singing

**DOI:** 10.3389/fnins.2020.00619

**Published:** 2020-06-16

**Authors:** Alessandro Dell’Anna, Jeska Buhmann, Joren Six, Pieter-Jan Maes, Marc Leman

**Affiliations:** ^1^Department of Musicology – IPEM, Ghent University, Ghent, Belgium; ^2^Department of Psychology, University of Turin, Turin, Italy

**Keywords:** joint action, embodied interaction, expressive quality, timing, Bayesian inference

## Abstract

Music is believed to work as a bio-social tool enabling groups of people to establish joint action and group bonding experiences. However, little is known about the quality of the group members’ interaction needed to bring about these effects. To investigate the role of interaction quality, and its effect on joint action and bonding experience, we asked dyads (two singers) to perform music in medieval “hocket” style, in order to engage their co-regulatory activity. The music contained three relative inter-onset-interval (IOI) classes: quarter note, dotted quarter note and eight note, marking time intervals between successive onsets (generated by both singers). We hypothesized that singers co-regulated their activity by minimizing prediction errors in view of stable IOI-classes. Prediction errors were measured using a dynamic Bayesian inference approach that allows us to identify three different types of error called fluctuation (micro-timing errors measured in milliseconds), narration (omission errors or misattribution of an IOI to a wrong IOI class), and collapse errors (macro-timing errors that cause the breakdown of a performance). These three types of errors were correlated with the singers’ estimated quality of the performance and the experienced sense of joint agency. We let the singers perform either while moving or standing still, under the hypothesis that the moving condition would have reduced timing errors and increased We-agency as opposed to Shared-agency (the former portraying a condition in which the performers blend into one another, the latter portraying a joint, but distinct, control of the performance). The results show that estimated quality correlates with fluctuation and narration errors, while agency correlates (to a lesser degree) with narration errors. Somewhat unexpectedly, there was a minor effect of movement, and it was beneficial only for good performers. Joint agency resulted in a “shared,” rather than a “we,” sense of joint agency. The methodology and findings open up promising avenues for future research on social embodied music interaction.

## Introduction

Music is a rewarding and empowering activity (Chanda and Levitin, 2013; Fritz et al., 2013), having the capacity to connect people (Malloch and Trevarthen, 2009; Overy and Molnar-Szakacs, 2009), and increase their self-confidence, their feelings of wellbeing, for example after singing together (Kreutz, 2014), and their motivation, for example in treating neurological disorders (Wan et al., 2010; MacDonald et al., 2012). While there exist other such facilitators of reward and empowerment, such as dance, ritual actions, sports, and other forms of joint actions (Sebanz et al., 2006), music is special in the sense that its social power is driven by auditory information, next to visual information. When you don’t see what the other is doing, your action can still be perfectly synchronized. And when muscles and brains get synchronized, strong group bonding effects may occur (McNeill, 1995). Obviously, while the use of music may date back to the very beginning of human evolution (Honing et al., 2015), its power is still working in all kinds of human social activities, including academic meetings, banquets, funerals, rituals, football matches, concerts, festivals, and so on.

Music can drive joint actions, intended as a “form of social interaction whereby two or more individuals coordinate their actions in space and time to bring about a change in the environment” (Sebanz et al., 2006: 70), and generate affects (e.g., of being in joint control and connected with others, see Keller et al., 2016 for a review). According to the minimal architecture hypothesis (Vesper et al., 2010), joint action can be investigated in terms of representations (the goal and tasks the subjects involved in it assign themselves), processes (prediction and monitoring of the various steps needed to accomplish it), and coordination smoothers (actions that simplify coordination). In this paper, we focus on timing prediction as a dynamic marker of the quality of a musical joint action in singing dyads and correlate it to subjective reports of that very quality and of the joint agency induced by it.

In recent studies, advantage has been taken of imposed musical tasks in order to understand how two or more subjects build representations of a joint action, employing both behavioral (Keller et al., 2007; Goebl and Palmer, 2009; Lesaffre et al., 2017), and neuroscientific (Arbib, 2013; Loehr et al., 2013; Keller et al., 2014) approaches to better understand music-based social empowerment (D’Ausilio et al., 2015). In a couple of works (Müller and Lindenberger, 2011; Müller et al., 2018), it has been shown that singing together (in a choir) implies also breathing and heart beating together, giving rise to a complex network of processes that, with the addition of body movements, imposes boundary conditions to its constituents (the singers), just like a “superordinate system.” In other words, singing together consists of a “participatory sense-making” that spreads out in the dynamics of the interaction itself, back to the subjects who get individually affected by certain properties of the singing (De Jaegher and Di Paolo, 2007; Schiavio and De Jaegher, 2017). Thereby, interaction cannot be understood by analyzing one single subject at a time, but rather by analyzing the interaction itself (in the form of behavior relative to one another).

However, at this point the question arises as to how good a music interaction should be in order for the music’s “bio-technological power” (Freeman, 2000) to become effective. A joint action such as singing can facilitate group-formation and generate feelings of connectedness, yet to what degree is this feeling depending on interaction qualities, that is, on the capacity to perform the rules as stated by the cultural context? In our opinion, few studies have addressed this question. Recent studies, indeed, use techniques that measure the bodily interaction of musicians mostly in view of timing, but quality as such is not addressed (Loehr et al., 2011; Eerola et al., 2018). To this effect, quality can be estimated through self-assessments by performers, or third persons. However, it is also of interest to consider the concept of joint agency. Pacherie (2012), in generalizing the concept of agency (Haggard and Eitam, 2015) from the individual to the group, distinguishes between a SHARED sense of joint agency and a WE sense of joint agency, pointing out that, if an action is a joint action, the resulting sense of agency should be a feeling of being in control of (at least) part of the joint action outcome. According to Pacherie’s distinction, people may experience a SHARED sense of joint agency in small groups, with a certain degree of specialization among the different participants, but without hierarchies, while a WE-agency might be experienced in larger ensembles with less specialization among its members and (sometimes) directed by a leader. Fitting examples are a volleyball team for the former kind, and a volleyball stadium choreography for the latter and, from a musical point of view, a small combo and an orchestra, respectively. In both cases, Pacherie stresses the importance of predictability, but to a different degree: a SHARED sense of joint agency may draw upon a low predictability of the partners’ action, whereas a WE-agency may draw upon a high predictability due to similarity among partners’ actions (what she calls “coordination symmetry”). Pacherie’s model has been successfully applied to dyads in studies comparing individual and shared control on a given joint action (Bolt et al., 2016; Bolt and Loehr, 2017). We extend this investigation so to include the WE-agency factor, trying to establish whether the (two, in our experiment) performers experience distinction from each other or blending into each other. The latter would imply a kind of boundary loss between agents.

To address the analysis of timing, we adopt a Bayesian inference framework to guide our methodological choices. Bayesian inference is the core approach behind the predictive coding theory (Vuust et al., 2009; Friston, 2010; Clark, 2016; Koelsch et al., 2019), a nowadays largely debated theory that sees the brain as an active generator of predictions, rather than a passive receptor of stimuli from the external world. Thanks to sensory feedback received in a continuous circular sensorimotor process, prediction errors are minimized for a given action the subject is about to accomplish. Brain networks thus formulate hypotheses about the possible state of the world (the prior) that are compared to the actual sensory information received (the error), in order to update the hypothesis (the posterior). Priors and posteriors are also called “beliefs,” not in the sense of explicit propositions, but rather in the sense of probability distributions, hence, mainly latent variables. A continuous updating of such beliefs is allowed by acting on the world in ways that minimize the error, or sensory surprise. Such sensorimotor loops, then, work as “active inferences” (Adams et al., 2013).

In a social context of music interaction, feedback on timing is provided by the other interacting subjects’ behavior. As stressed by Koelsch et al. (2019), music is a perfect case against which the predictive model may be tested, because the music’s very syntactic structure implies rhythmic, melodic and harmonic expectancies, that is, prediction. We believe that accurate prediction of the partner’s action is crucial in musical ensembles, even if it shows in different degrees, depending on musical genres, cultures, and kind of ensembles (Rohrmeier and Koelsch, 2012; Salimpoor et al., 2015). In this paper we focus on timing since this is one of the main features in which the quality of music is reflected, and probably the most tractable with our approach. The Bayesian inference framework is here used to develop a computational analysis that copes with prediction errors in conditions where timing can be unstable, thereby assuming that performers construct latent time-varying beliefs about their joint timing. Our quest for interaction quality is therefore also a quest for a proper methodology for estimating latent variables about joint timing. Importantly, such a timing marker has to be considered as a dynamic index of coordination insofar as it takes into account not only the timings of the two musicians separately and correlate them afterwards (for example, by means of windowed cross-correlation), but several inter-onset intervals constituted by the two interacting musicians’ singing (see section “Materials and Methods”). It is worth stressing that, while timing errors may be due to a variety of factors (inability of reading the notes, general lack of ability to accurately follow to synchronize singing with beat, etc.), in this paper we are interested in the dynamics, rather than the causes, of timing quality.

Following the growing interest in embodied approaches to cognition (Thompson and Varela, 2001; Gallagher, 2005; Chemero, 2009), in particular music cognition (Iyer, 2002; Leman, 2007; Walton et al., 2015), the kinaesthetic dimension of musical performances has been widely explored in the last years, stressing the impact of body movements on both production and perception. The predictive coding framework, combined with these embodied approaches (Gallagher and Allen, 2016), can help explain how movement, along with other sensory modalities, could contribute to error minimization. Indeed, when body parts move in time with the music, their timing reflects the timing of the music and can help shape this timing during production (be it singing or playing an instrument, see Wanderley et al., 2005; Maes et al., 2014).

**FIGURE 1:**
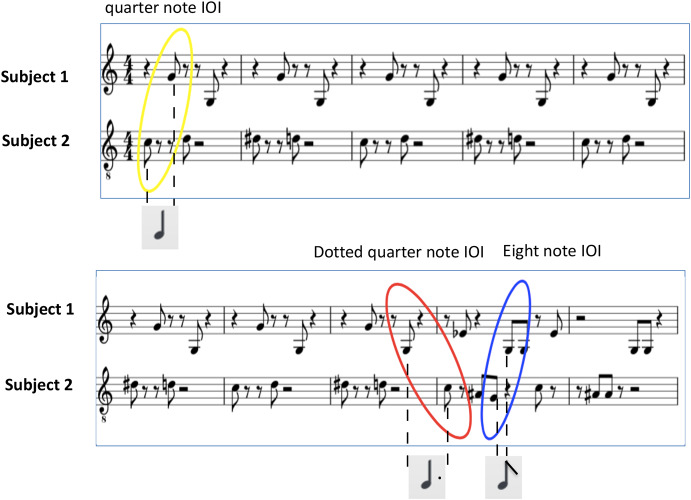
Experimental stimulus. Part of the participants’ scores. Together these scores form a semi-hocket, meaning that there are no simultaneous notes, and the combined scores merge into one melody. This melody is an adaptation of Michael Jackson’s Billy Jean. The two parts contain an equal number of notes, displaying the same level of difficulty. In yellow, red and blue the three IOIs used in Bayesian regression (see below) are highlighted.

In the present study, we explore interaction quality in a singing dyad, taking advantage of the medieval “hocket” style, in which two (or more) musicians are required to build a melody together using strict alternation of notes. Our hypotheses are as follows:

1. The quality of music interaction is reflected in the timing of the joint action among performers. Performers can estimate their own interaction quality through continuous video annotation (video-stimulated recall) and they can assess the social effect of the interaction in terms of an assessment of their joint agency experienced during the interaction. As quality is reflected in timing, joint action timing can be measured as the performers’ latent (or emerging) belief about joint timing. We predict that more accurate timing is correlated with higher quality, as reflected (i) in the performers’ higher self-annotation of their own performed interaction quality, and (ii) in the performers’ estimation of joint agency experiences.
2. Given the high similarity of the singers’ music score (see Figure 1), a high quality in performing will correspond with a high sense of joint agency values, that is, by WE-agency.

3. Movement may help performers to make their timing more accurate. Indeed, since multiple senses take away uncertainty (according to the predictive coding theory) and movement is timing (according to embodiment theory), movement should affect quality.

## Materials and Methods

### Ethics Statement

Participants were informed in advance about the task, the procedure and the technology used for measurement. They had the opportunity to ask questions and were informed that they could stop the experiment at any time. The ethics committee of the Faculty of Arts and Philosophy of Ghent University approved the study and the consent procedure.

### Participants

Fifteen couples of musicians were recruited (mean age 29.4 ± 10.4 years; 12 women), both participants being either men or women, so that their pitch range could match more easily. Only couples for which both participants knew one another were considered, in order to reduce performance stress for an intimate task such as singing. As musicians we considered people currently playing an instrument (or singing) with at least 5 years of regular (formal or informal) musical training (mean 10.1 ± 9.7), capable of singing a simple melody from sheet music.

### Task

In this experiment we let pairs of musicians sing “on stage” an interleaved melody provided on a score. They were told that their parts should never overlap, and that the combination of the parts would result in a melody consisting of an A- and a B-part. They were also instructed to try to keep going on with singing even if for some reason their interactive performance would break up. We asked the participants to sing the notes by producing the sound “ta” or “pa.” The fact that these sounds start with a plosive facilitates automatic onset detection of sung notes, needed to extract inter-onset-intervals (IOIs). In hocket polyphonic style a single melody is broken down into two or more parts that never overlap, alternating almost regularly one tone after another. Here we use a semi-hocket technique for two singers, where alternation is somewhat less strict, meaning that sometimes a singer might sing two notes in a sequence (see the music score, Figure 1). We assume that the quality of the singing is reflected in the performers’ timing of the sung notes, in particular also in the joint timing of the sung notes. In a good performance we expect the relative timing of the notes to correspond to the relative timing of the notes in the score, whereas a bad performance would contain note durations that do not correspond with those of the score. Due to a limited rehearsal time (5 min alone, 15 min together) the task was expected to be challenging, leading to different outcomes in performance quality. After the rehearsal, singers had to perform eight trials of two randomized conditions lasting two minutes each, either moving (four trials), or not moving (four trials). In the non-movement trials participants were asked to stand as still as possible, while performing the singing task. In the movement trials participants were invited to move as they pleased while performing. This could result in simple hand- or foot-tapping, head-nodding, body-swaying, or even dance-like movements.

### Technical Setup

**FIGURE 2:**
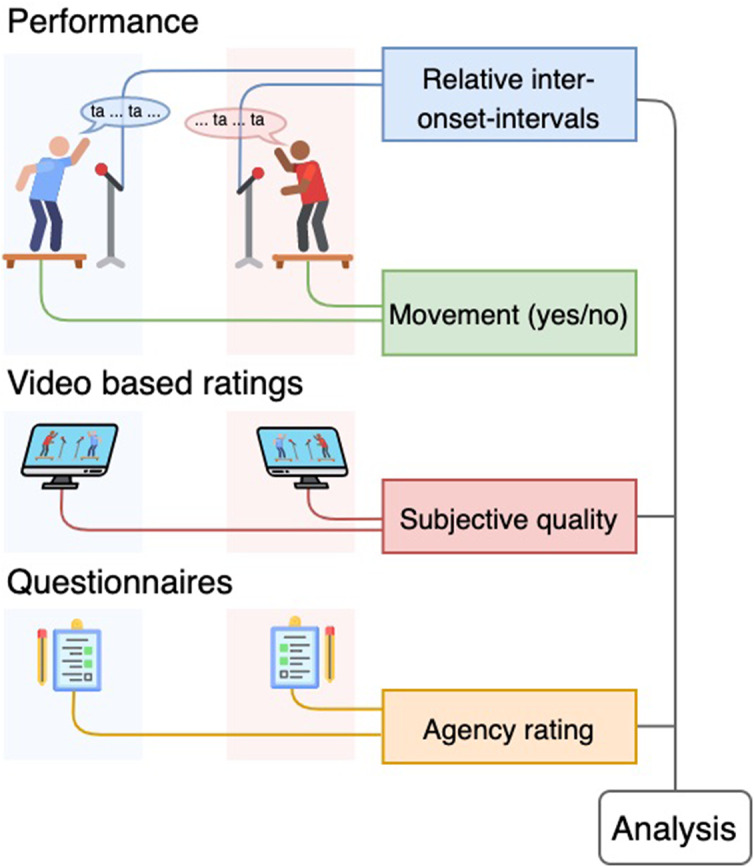
Experimental procedure. Each experiment has three parts. The performance is followed by video based ratings and questionnaires. During performance the participants are allowed to move or not, which is checked by the movement outcome. Other measures are combined in the analysis.

For each recording of the musical interaction task the two participants were standing on a force plate facing each other (see Figure 2). Both force plates have four weight sensors at the corners in order to register movement of the participants. The measured voltages are converted to MIDI control change (CC) messages by means of a Teensy 3.2 microcontroller. The MIDI stream was recorded in Ableton 9. The encoding of sensor signals into MIDI makes it straightforward to record audio and sensor data in sync using standard DAW software such as Ableton. A decoder script turns the MIDI into data fit for analysis. Participants were equipped with a headset containing a small microphone. The singing was thus captured and also recorded with Ableton. In addition, a video recording was made with a webcam (Logitech, c920). The webcam was modified to allow audio input. The audio input is connected to a SMPTE source to synchronize the video with the audio. These audio-visual recordings were used immediately after the recording session. The participants were requested to review their performances and annotate the quality level of their interaction. This was done via a script that synchronized and merged the audio and video recordings per trial. The scoring of the interaction happened on two separate computers via the mouse that could move a visual line up (better quality) and down (worse quality). The visual scores are stored as thousand samples of values between 0 and 127. The initial position of the cursor was set to value 64, a neutral starting point. All recording devices were connected with a master sync clock (Rosendahl Nanosyncs HD), preventing drift and enabling precise synchronization of audio movement, and video data.

### Procedure

Each couple was welcomed in our laboratory and, after filling in the informed consent, participants were explained that they had to build a melody together, combining their individual parts, stressing that these should never overlap, but almost always alternate one note after the other. Moreover, subjects were not allowed to read their partner’s score. Then, they rehearsed their part in two separate rooms for 5 min, having the opportunity to listen to it once or twice in order to find the right pitch and learn the melody. Afterwards, they were gathered in the main lab, equipped with the headsets, and invited to get on “the stage,” that is, on the two force plates facing each other at 1.5 m, to rehearse together for 15 min maximum, before the beginning of the real performance. After recording the eight trials, each participant individually executed a quality assessment task concerning the performance and the sense of joint agency (see below), without communicating with the partner. In total the experiment took between 1.5 and 2 h per couple.

### Data Pre-processing

#### Audio Onset-Detection

As our approach has a focus on the timing of the singing, the audio recordings are reduced to the onsets of the singing, by doing an automatic onset detection in *Sonic Visualizer*, followed by a manual checking and correction step, that involved adding onsets in case they were not automatically added, and/or removing automatic onsets that did not accord with a sung note. The onsets are then converted into IOIs, that is durations that mark the time between two successive onsets (whoever sung them). The analysis is thus based on relative IOIs, that is, the IOIs formed by both performers. According to the score, a performance should result in three types of IOI durations, matching the durations of eighth notes, quarter notes, and dotted quarter notes (Figure 1). Depending on the tempo, a 2-min performance equals approximately singing the A- and B-parts four times. In theory this would result in 176 eighth notes, 96 quarter notes, and 47 dotted quarter notes.

### Subjective and Objective Markers of Interaction Quality

#### Annotation and Questionnaires (Subjective)

The two participants of each couple were separately asked to assess the general quality of the interaction, that is, the performance as a whole, rather than the quality of their individual performance or the other participant’s performance. This resulted in two time-series of quality values between 0 and 127 for each trial. Secondly, participants were asked to assess the joint sense of agency on a 7-point Likert scale. In particular, for each of the eight trials the subjects were asked to answer the question “When looking at the moments with the highest quality assessment, how was your feeling of control over the process on a scale between 0 (independent), 3 (shared), and 6 (complete unity with your partner)?” We explained this question by saying that the interaction could be either the product of two actions not really well coordinated between them (independent) or the product of two coordinated but distinct actions (shared), or the product of two actions that are not felt as different, but rather as the accomplishment of a single subject (unity or WE-agency).

#### Third-Person Quality Assessment (Subjective)

As expected, we observed differences in the performance quality of the different couples. Given the fact that there was a large variation in performance quality, the authors of the paper agreed upon a subjective classification of the performances per duo into two groups, i.e., expert group and non-expert group. This was done by looking at the performance videos and evaluating the stability of the performance (could couples keep up their performances without too many break-ups) and how similar the performance was to what was written in the score. Six couples were assigned to the non-expert group and nine couples to the expert group. This subjective classification was done to validate our assumption that a good performance has less errors in the timing of the singing than a bad performance (Figure 4).

#### Performance Timing Errors (Objective)

The score defines a musical norm for interactive performances, including rhythmic figures, tempo and an overall melodic narrative. However, due to the fact that the music emerges from the interaction, we assume that singers predict each other’s performance in order to perform their own contribution correctly. As mentioned, not all performances may reach a high-quality level of interaction. Given the constraints of the musical rules, we consider three different types of prediction-errors, related to:

• *Fluctuation*: The fluctuation errors are defined as micro-timing (in milliseconds) prediction-errors that result from different sources such as timing-corrections due to small mistakes, due to active sampling, or even small onset measurement errors within the data pre-processing. Overall, fluctuation is a source of variance that can be considered necessary in order to maintain a stable performance state, even of high quality.
• *Narration*: The narration errors are defined as meso-timing (typically up to half a second, related to note durations) prediction-errors that may occur when a performer fails to follow the musical rule, for example, by forgetting a note, or making a mistake in note duration. Pitch is not taken into account, only timing. Overall, an error in the sequence (for example due to the omission of a note) may disturb the ongoing interaction. However, the dynamic system may be resilient enough to recover from such errors.
• *Collapse*: The collapse errors are defined as macro-timing (up to several seconds) prediction-errors that may occur when the performance, hence also the musical interaction, breaks down. The breakdown is catastrophic in the sense that both performers lose control of the expected musical narrative. This error is different from the narration errors that allow recovery due to resilience. To recover from such an interaction collapse, it may be necessary to start a new narrative from the beginning of the piece or the beginning of a section.

### Data Analysis

#### Bayesian Inference Approach

**FIGURE 3:**
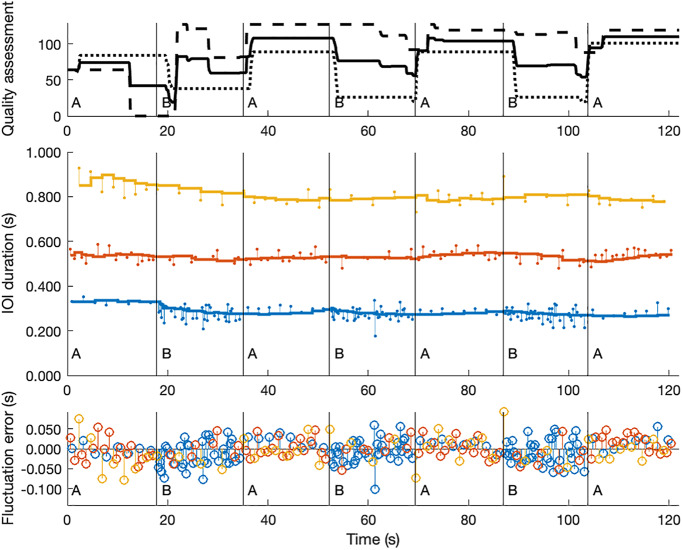
Performance interaction measurements in time-series. In this figure, three time-series of the same performance (a 2-min trial) are visualized. The top plot shows the two quality assessments of the two participants (the dotted lines); the solid line represents the mean of the two assessments. The horizontal lines of the middle plot show how the priors of the three IOI classes evolve over time, according to our Bayesian sequential updating approach. The vertical lines with small dots indicate the deviations of the actual IOI durations with respect to those priors; in other words, they represent the errors in seconds. The plot at the bottom is a summary of the middle plot, where the zero-line represents the priors and the vertical lines are the errors (in seconds) for the three IOI classes (blue = short IOIs; orange = middle IOIs; and yellow = long IOIs).

As our data-analysis approach is based on the idea that performers try to reduce performance errors with respect to predictions, we consider performers as components of an interaction dynamics. We assume that each performer makes a prediction of the timing of the joint action (the interaction) based on a latent, or emergent, variable that estimates the timing of the relative IOIs in terms of milliseconds. As the piece contains only three different IOI classes, we assume that performers construct a latent variable for the estimated timing of each IOI-class. Obviously, the timings of the IOI classes are mutually constrained, thus contributing to a global latent variable, which is known as tempo. In our analysis we focus on how performed IOIs relate to the latent IOI-classes. Rather than inferring the prediction errors from an estimated global tempo (and proportional ratio of that tempo with respect to the IOI-classes) our method is tolerant to a systematic shortening or lengthening of IOI-classes according to performers’ expressive timing preferences. The initial values of the variables that estimate the timing of the IOI-classes are set by a *k*-means clustering on all IOIs in three IOI-classes, using the first 15 s of a performance. Thereafter, a sequential Bayesian updating is performed for each of the IOI-classes separately, using a 15-s window of incoming IOI values (leading to the evidence distribution or the likelihood of measurement). Using Bayesian terminology, we interpret the prior as the mean of a distribution of old predicted durations of the IOI-class and the posterior as the mean of an updated distribution due to new evidence. This procedure is executed step by step (i.e., one IOI after another in the time series). It allows us to calculate the difference between the performed IOI and the predicted IOI, in milliseconds (Figure 3). For the entire performance, we calculate the root-mean-square error (RMSE) for each IOI-class, and take the average over all IOI-classes. This approach can deal with small changes in tempo and therefore, it accounts for the assumption of non-stationarity. In fact, for each IOI-class we use proportional timing errors by taking the log2 of the ratio of the measured IOI and the predicted IOI.

While the above approach may be working for fluctuation errors, we also have to consider the fact that IOIs may be wrongly classified due to narration errors. In order to account for these narration errors (which are restricted to duration errors), we keep track of the sung (duration) sequence and the expected corresponding IOI-class assignments. When expected IOIs get wrongly classified they are considered as narration errors, expressed in percentage of matching IOIs. Collapse errors are considered to be larger gaps in the performance (IOI durations that differ more than two standard deviations from the corresponding IOI-class prior), where normally onsets would have been expected. The collapse errors are expressed as a percentage of the number of collapses compared to the total number of IOIs in the performance.

#### Correlations Between First-Person Viewpoints and Timing Errors

The correlation was calculated between the overall timing errors per trial and the average joint-agency scores that were indicated in the questionnaires. This was done for each performance-error type. Since the joint agency scores are not normally distributed and they contain a lot of identical values (7-point Likert scale), Kendall’s Tau correlation was used. In a similar manner the correlation between timing errors and the quality assessment scores was calculated.

#### The Effect of Movement and Expert Group on Performance Errors

In order to test our hypothesis that movement has an auxiliary function in error minimization during a joint singing task, for each type of performance error (fluctuation, narration, and collapse) we compare the average error value of the four movement trials with the average of the four non-movement trials. A 2 × 2 mixed ANOVA was performed with condition (movement/non-movement) as within-subject factor and expert-group (yes/no) as between-subjects factor. In a few cases the performance errors in a group were not normally distributed. When data distributions were not normal or the assumption of homogeneity of variance was violated, non-parametric tests were executed instead (Wilcoxon signed rank tests to compare movement with non-movement condition for each expert level).

#### The Effect of Movement and Expert Group on Agency and Quality Assessment

To validate our hypothesis that movement and expert level have a positive impact (higher agency and quality scores for experts, while moving) on the subjective assessment of a performance interaction, a 2 × 2 mixed ANOVA was performed. Identical to the test on performance errors, condition (movement/non-movement) is the within-subject factor and expert-group (yes/no) the between-subjects factor.

#### Movement Assessment

For each trial, continuous wavelet transforms were performed on the movement data of the two force plates, i.e., for each force plate the sensor that captured the highest amplitude. Only the wavelet information within the movement-relevant range of 0.25 to 5 Hz was considered. Within that range the frequency band with the highest average wavelet magnitude was selected. For each force plate this average magnitude was used to calculate the average movement magnitude for the couple. The right-skewed histogram of these values for all the non-movement trials covers a small range of magnitude values, with a maximum average magnitude value of 11. For the movement trials the histogram covers a much wider range of magnitude values, with a maximum of 88. In accordance with what was observed in the video recordings, a threshold of 25 was chosen as the cut-off for detected movement (above), or not (below).

## Results

### Effect of Movement and Expert Group

#### Performance Timing Errors

In total, nine out of the 120 performance trials (7.5%) were excluded from analysis. Two trials were excluded (the first and third trial of duo 5), because the participants made a lot of errors by singing (almost) simultaneously, resulting in IOIs that were too short to be valid eighth note durations. In other words, in these two trials the participants did not perform the singing task as described in the musical score. Seven more trails were excluded (duo 5, trial 2; duo 12, trial 1; duo 13, trial 2 and 4; duo 18, trial 3; duo 19, trial 5; and duo 21, trial 7), because too much movement was detected in the conditions where participants were instructed not to move, i.e., the continuous wavelet transforms of the sensor-data coming from the force plates revealed magnitude values that were higher than the defined threshold for non-movement.

Fluctuation errors are not significantly lower in the movement condition (*M* = 0.185, *SE* = 0.022) than in non-movement condition (*M* = 0.217, *SE* = 0.036), *F*(1, 13) = 3.929, *p* = 0.069, and *r* = 0.48. There was a significant effect of expert level, indicating that experts had lower error rates (*M* = 0.133, *SE* = 0.012 for movement; *M* = 0.147, *SE* = 0.013 for non-movement) than non-experts (*M* = 0.245, *SE* = 0.036 for movement; *M* = 0.298, *SE* = 0.064 for non-movement), *F*(1, 13) = 7.938, *p* = 0.015, and *r* = 0.62. No significant interaction effect was found between movement and expert level, *F*(1, 13) = 1.436, *p* = 0.252, and *r* = 0.32.

Narration matching in the movement condition (*M* = 84.54, *SE* = 3.43) is not significantly different from that in the non-movement condition (*M* = 82.90, *SE* = 3.69), *F*(1, 13) = 1.437, *p* = 0.252, and *r* = 0.32. There was a significant effect of expert level, indicating that experts had higher percentages of predictable IOI classes (*M* = 94.92, *SE* = 1.44 for movement; *M* = 93.18, *SE* = 1.98 for non-movement) than non-experts (*M* = 72.67, *SE* = 3.46 for movement; *M* = 71.15, *SE* = 4.45 for non-movement), *F*(1, 13) = 31.913, *p* < 0.001, and *r* = 0.84. No significant interaction effect was found between movement and expert level, *F*(1, 13) = 0.007, *p* = 0.937, and *r* = 0.02.

For collapse errors, a Wilcoxon signed ranks test revealed that for non-experts a significantly higher percentage of collapses occurred in the movement condition (*Mdn* = 8.66) than in the non-movement condition (*Mdn* = 6.18), *z* = −2.366, *p* = 0.018, and *r* = −0.43. However, for experts the percentage of collapses were not different for moving (*Mdn* = 0.45), and not moving (*Mdn* = 0.45), *z* = −0.700, *p* = 0.484, and *r* = −0.13.

#### Joint Agency and Quality Assessments

With respect to agency, a Wilcoxon signed ranks test revealed that for non-experts, agency ratings were significantly higher when moving (*Mdn* = 3.28) than when not moving (*Mdn* = 2.63), *z* = −2.043, *p* = 0.041, and *r* = −0.39. However, for experts, agency ratings were not significantly different for moving (*Mdn* = 3.88), and not moving (*Mdn* = 3.13), *z* = −0.762, *p* = 0.446, and *r* = −0.14.

The quality assessment in the movement condition is not significantly different from that in the non-movement condition, *F*(1, 13) = 1.880, *p* = 0.194, and *r* = 0.36. There was a significant effect of expert level, indicating that experts gave higher annotation scores (*M* = 89.70, *SE* = 4.90 for movement; *M* = 83.99, *SE* = 5.90 for non-movement) than non-experts, (*M* = 69.03, *SE* = 5.56 for movement; *M* = 63.70, *SE* = 3.54 for non-movement) *F*(1, 13) = 11.477, *p* = 0.005, and *r* = 0.68. No significant interaction effect was found between movement and expert level, *F*(1, 13) = 0.002, *p* = 0.962, and *r* = 0.01.

### Correlations of Performance Timing Errors

All types of performance error are significantly correlated with one another. Table 1 shows the correlation values and their corresponding significance values for the three types of performance errors.

**TABLE 1:** Performance error correlations.

	**Fluctuation vs. narration**		**Fluctuation vs. nollapse**		**Narration vs. nollapse**	
***Trials***	***τ***	***p***	***τ***	***p***	***τ***	***p***
All	−0.67	<0.001	0.46	<0.001	−0.52	<0.001
Movement	−0.60	<0.001	0.50	<0.001	−0.57	<0.001
No movement	−0.75	<0.001	0.46	<0.001	−0.50	<0.001

### Correlations of Performance Timing Errors With Agency and Quality Assessments

Fluctuation errors are negatively correlated with agency assessments, although correlation values are low (*τ* = −0.15). The lower the fluctuation error, the higher the agency assessment value. Narration is positively correlated with quality assessments. The higher the percentage of predictable IOI classes, the higher the agency assessment value. Collapse errors are negatively correlated with quality assessments. The lower the percentage of collapses, the higher the agency assessment value.

With respect to the quality assessments, higher correlations are found. Fluctuation is negatively correlated with quality assessment. The lower the fluctuation error, the higher the quality score. Narration is positively correlated with quality assessments. The higher the percentage of predictable IOI classes, the higher the quality assessment value. Collapse errors are negatively correlated with quality assessments. The lower the percentage of collapses, the higher the quality assessment value. Table 2 shows all Kendall’s tau correlation coefficients and the corresponding significance values.

**TABLE 2:** Performance error correlations with subjective performance assessments.

		**Fluctuation**		**Narration**		**Collapse**	
	***Trials***	***τ***	***p***	***τ***	***p***	***τ***	***p***
Agency	All	–0.15	0.028	0.18	0.010	–0.24	0.001
	Movement	–0.10	0.311	0.17	0.083	–0.31	0.002
	No movement	–0.16	0.126	0.22	0.030	–0.26	0.013
Quality	All	–0.37	< 0.001	0.40	< 0.001	–0.41	< 0.001
	Movement	–0.35	< 0.001	0.35	< 0.001	–0.48	< 0.001
	No movement	–0.35	< 0.001	0.43	< 0.001	–0.36	< 0.001

## Discussion

The present paper investigated whether the quality of interaction, while performing music, plays a role in the establishment of joint action and group bonding experiences. The hypothesis that interaction quality plays a role was tested with singing dyads. We thereby focused on timing markers. We achieved three main outcomes. Firstly, we found correlations, albeit weak, between the sense of joint agency and measured fluctuation, narration and collapse errors. Contrary to our prediction, the highest degrees of joint agency reached by the dyads point to a SHARED rather than a WE sense of agency, particularly in the movement condition. Secondly, we found correlations between the self-annotated performance quality and measured fluctuation, narration and collapse errors. Although movement as such did not produce overall improvement in the quality of the performances, we observed a tendency for participants to reduce fluctuation errors while moving. On the contrary, non-expert dyads showed more collapse errors in that condition. These results point toward a different kind of effect of movement on micro- and macro-timing: when movement might possibly reduce micro-timing errors in general (recall that the difference was close to significant, with a medium effect size), it disrupts the performance on a macro-timing level for less experienced performers. Finally, we contributed to a novel and effective methodology and framework to analyse the objective quality of the interaction from the point of view of timing.

An important limitation of our study concerns the fact that the assessment of the feeling of joint agency was done after watching the performance recording, while moments of joint agency are supposed to occur during the performance. The correlation results are promising, but they point toward the need for a more refined method for estimating agency. The idea of using a hocket composition in our study was also inspired by Bolt et al. (2016)’s discovery that sequences of (twelve) tones played at the piano by pairs of non-musicians, first by one subject and then by the second one, resulted in lower values of joint sense of agency compared to when the subjects alternated a tone after another. Moreover, these authors found that objective coordination between the subjects (measured by means of cross-correlation of the tones’ onset series) impacted on joint agency, enhancing it when the coordination was strong. These findings are coherent with ours, though the data were obtained with different analytical tools and in a study that did not deal with expressive quality. In the present paper, we were also interested in the kind of joint agency such a performance could induce. Therefore we administered a questionnaire asking the subjects an assessment of their experienced sense of joint agency, stressing that the lowest values indicated an independent control, the medium values a shared control and the highest values a complete unity with the partner in controlling the musical joint action. Since the average collected values were in the medium range, our results point toward a SHARED rather than a WE sense of agency. This outcome complies, indeed, with Pacherie’s definition of the two kinds of joint agency (Pacherie, 2012), in particular when she suggests that a SHARED agency would ensue from a small group joint action, in which roles can be easily distinguishable. At this moment, we can speculate that this feature overcame the high similarity we intentionally established between the two scores. Indeed, according to Pacherie, the high predictability of, and, as a consequence, low necessity to keep oneself distinguishable from, the partner could have caused a WE-agency, rather than a SHARED agency (see Fairhurst et al., 2013 for a similar idea and some neuro-scientific possible account of it). Sticking to this result, we may then conclude that, on average, our musical task did not induce any boundary loss between the subjects in the pair, but we cannot exclude that the difficulty of the task contributed to prevent it. All in all, this finding adds to the debate on joint agency not only in musicology, but also in the wider domain of cognitive science (van der Wel et al., 2012; Dewey et al., 2014; Bolt and Loehr, 2017).

**FIGURE 4:**
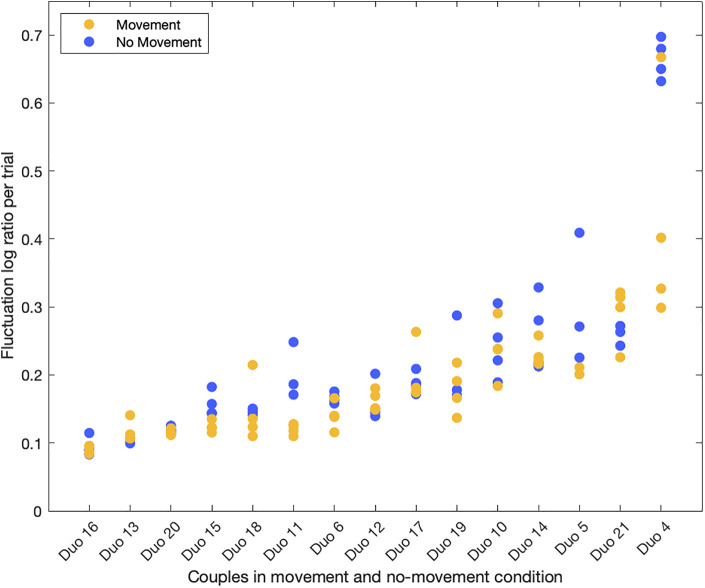
Fluctuation errors per duo. The duos are ordered from smallest to largest average fluctuation error over the eight performance trials.

Subjective self-annotations of the quality of the performance have to be treated carefully as well. The correlation results are promising and they seem to indicate that performance quality can be self-assessed in a proper way, although improvements to our slider approach in the video-stimulated recall protocol are still possible. Here, an important limitation of our study consisted in the latency between the recorded performance and the annotation the subjects did by means of the slider, meaning that the assessment cannot match perfectly the moments it refers to, but it is always a bit late. Furthermore, we asked the subjects to assess the quality of the performance as a whole, without focusing on timing, since we were interested also in other expressive features like pitch and tuning (whose analysis we are bracketing in the present study). Yet, given the crucial role of timing in music and its capacity to create social bonding in synchronization tasks (Hove and Risen, 2009; Wiltermuth and Heath, 2009; Kokal et al., 2011), we assumed timing was the main feature to be analyzed in our study. The good level of musicianship declared by our subjects, and visible in many of their performances, should bolster the validity of the correlation we found. Of course, not all couples reached the same quality levels, as it is manifest from both the objective and subjective measurements and from Figure 4, which shows the clustering of each couple’s trials according to their fluctuation errors. Yet, we think that considering the relationships between those measurements gave us some hint about a proper treatment of the expressive quality in a singing dyad.

Also, the relatively large number of rejected data may induce some improvement of our paradigm. Indeed, most of the rejected trials were due to the fact that subjects did not comply with the experimental condition, either moving when they were supposed not to do so or singing completely different than what was in the score. Some kind of feedback, either a visual or an auditory feedback, could inform the subject about his/her passing a given movement threshold, thus allowing to adjust for it. After all, both visual and auditory bio-feedback systems may be conceived of in order to adjust the performance itself according to the amount of (mainly fluctuation and narration) errors collected in a given time interval. This is how we see a relevant application of our method aiming at enhancing musical learning processes (see Moens and Leman, 2015, for some applications of the same principle to running and walking to the music).

In this paper we developed a novel methodology to capture the interaction of a singing dyad. While the method was applied to the emergent timing of both singers, the method allows an analysis of each singer separately, despite possible changes in tempo. In accordance with the recent emphasis on the predictive coding framework (Friston, 2010; Clark, 2016), also in music studies (Vuust et al., 2009; Koelsch et al., 2019), we applied a Bayesian inference approach to dynamically analyse a semi-hocket interaction between two subjects. In fact, a singing dyad can be conceived of as a dynamical system whose components constrain each other’s unfolding performance (Müller and Lindenberger, 2011; Konvalinka and Roepstorff, 2012; Müller et al., 2018), considering its variability and correcting for it, when needed. A sequential Bayesian process allowed for an analysis in the form of a continuous updating of timing-error minimisation. We focused on timing and identified fluctuation, narration and collapse errors as objective, third-person markers of the quality of a musical interaction, exploiting the idea that the “superordinate system,” i.e., the dyad, rather than the single singer, constructed predictions of latent variables that keep track of the timing of each relative IOI. This approach has the advantage that we look finer in time than a method that would focus on the overall tempo. Obviously, it can be questioned whether this construct has any psychological plausibility, yet the emergence of latent variables is a known phenomenon, and in full agreement with the predictive coding approach. For example, the concept of latent variables that work as predictors for observable/measurable action can be compared with the two processes postulated to correct errors in a sensorimotor synchronization task at the individual level, phase correction and period correction, the former being an almost automatic process with which fluctuation errors can be equated, the latter requiring a conscious effort comparable to the one needed to overcome narration errors (Wing et al., 2010; Repp and Su, 2013). The distinction between fluctuation, narration and collapse errors was introduced in order to deal with typical performance errors. Fluctuation may be related to subconscious active sampling in order to be able to update the latent variable on timing. Further research is needed to refine its sources of variability. Narration relates to a symbol-based account of the performance and therefore, we assume that it has a cognitive origin related to memory and sequencing. While collapse errors induce a complete breakdown of the performance, the singers may still cope with narration errors (possibly with period correction), even if they surely threaten the quality of the performance. We believe that the Bayesian inference framework offers a useful method for assessing musical expression in high quality music performance. As our concept is based on relative IOIs, the method offers the perspective that it can be applied to groups comprising three and more singers and musicians.

Finally, movement did not improve the performance timing, but the fact that the worse couples made more collapse errors in the movement condition, along with the higher joint agency values reported in that condition and a tendency for all participants to reduce their fluctuation errors in that condition, suggests that above a certain level movement may impact on the overall quality of the performance. In particular, this result could imply that, while for bad couples movement constitutes an interference with their task, good couples may benefit from it at a micro-timing level. This hypothesis is compatible with a Bayesian approach insofar as bad couples, by definition, find it difficult to both coordinate their movements with the music and their singing with the partner’s, that is, predicting the music and the partner at the same time. On the other hand, active inference may be enhanced by moving for those couples that are already fluent, but can take further advantage from moving at a micro-timing level. However, further research is surely needed to better disentangle the network of dynamic processes that is constituted by prediction, agency and movement in musical expressive moments (Leman, 2016).

As far as we know, this is the first study that applies principles of the predictive coding approach to a social musical interaction. And it does so by stressing the dynamic character of the interaction thanks to a parameter, the relative IOI, which treats two subjects as one, hence taking seriously the Gestalt concept that the whole is more than the sum of its parts. The same idea is implicit in the concept of participatory sense-making (De Jaegher and Di Paolo, 2007), which emphasizes that the sense of a joint action is not given in advance, but it is co-constituted by the interactive subjects. In a musical context, thereby, the musical object is not constituted either by the score or by the representations in the minds of each musician, not even by the auditory event in itself, but rather by the embodied interaction of the musicians on the fly (Schiavio and De Jaegher, 2017). The focus on the interaction, rather than on the single components of it, increases the complexity of studying an already complex phenomenon like music, although also in the domain of cognitive neurosciences several appeals have been recently made toward such a perspective change. For example, Schilbach et al. (2013) write that “After more than a decade of research, the neural mechanisms underlying social interaction have remained elusive and could – paradoxically – be seen as representing the ‘dark matter’ of social neuroscience” (ibidem: 394). Hyper-scanning, the simultaneous acquisition of cerebral data from two or more subjects, is a promising technique to approximate such an ambitious aim (Konvalinka and Roepstorff, 2012; Babiloni and Astolfi, 2014). Indeed, though not yet analyzed, not only did our experiment carry out a motion capture collection of data from the singing dyads, but it also planned the physiological recording of skin conductance by means of portable bracelets. Moreover, we are working exactly on the possibility to simultaneously electroencephalography (EEG) recording two interacting musicians, in search of the brain basis of social embodied music interaction. Such an empowered set-up would likely allow both to test the psychological plausibility of a dynamic marker of timing as the one we devised in the present paper and to identify possible dynamic neural markers of timing and other musical features and processes (see also Osaka et al., 2015; Nozaradan et al., 2016; Pan et al., 2018). Ultimately, such enterprise would probably require a thorough theoretical synthesis between embodied and predictive approaches to (music) cognition, of which the present work can be seen as a first empirical application.

## Data Availability Statement

The datasets generated for this study are available on request to the corresponding author.

## Ethics Statement

The studies involving human participants were reviewed and approved by the Ethics Committee of the Faculty of Arts and Philosophy of Ghent University. The patients/participants provided their written informed consent to participate in this study.

## Author Contributions

AD developed the experimental design, executed the experiment, and wrote the manuscript. JB executed the experiment, analyzed data, and wrote the manuscript. JS build the technical set-up for the experiment. P-JM contributed to the analysis and technical set-up building. ML conceived the analysis and contributed to write the manuscript.

## Conflict of Interest

The authors declare that the research was conducted in the absence of any commercial or financial relationships that could be construed as a potential conflict of interest.
